# Augmenting neurocounseling in addiction recovery with intelligent systems: a roadmap for research and practice

**DOI:** 10.3389/fpsyt.2026.1752535

**Published:** 2026-06-10

**Authors:** Christopher Ashton, Denise Duffie

**Affiliations:** 1 TeDDs on Chapel, Center for Recovery, Grand Sault, NB, Canada; 2 Beal University, Bangor, ME, United States

**Keywords:** acceptance, addiction-computational neuroscience, cognitive impairment, community, counseling, intelligent system, recovery

## Abstract

Community and counseling resources continue to support persons and families affected by addiction. Knowledge of the neuroscience underlying addiction continues to rapidly emerge and is beginning to influence care methods. The complexity of the neurobiology remains difficult to definitively associate with symptomatology of persons engaged in recovery. Assessment and appropriate evidenced based methodologies remain challenging for practitioners and clients both. Neurocounseling has been proposed as a promising practice to elevate the effectiveness of professional care. This paper outlines the current known variables involved in the behavioral neuroscience of addiction with a focus on the science of recovery. Intelligent systems using least intrusive methods are proposed to refine cause, effects, timelines and counseling methods to better support addiction counseling.

## 1 Introduction

The challenge of initiating and sustaining remission from substance use disorder is well documented. While pharmacotherapies, psychotherapies, and peer recovery programs can be effective, relapse remains common, and overdose risk remains significant. Many individuals face confusing and fragmented options in seeking appropriate help. The 12-step tradition continues to support millions of people through freely available meetings and relationships. In parallel, clinical neuroscience has deepened the understanding of habit learning, salience attribution, cognitive control, stress, and social reward systems that contribute to addiction and recovery.

In a recent work, we proposed a neurocounseling paradigm centered on the principles of Step One of 12-step programs, which involves acceptance of powerlessness over substances and unmanageability of life (1). That paper argued for a science of early commitment to abstinence as exemplified by action, not as a narrow doctrinal claim, but as an integration of the biological, psychological, social, and spiritual processes that co-occur when change becomes possible. The aim was to build a bridge between the available predictions of neuroscience and the lived wisdom of community and counseling practice.

By neurocounseling, we refer to the practice of neuroscience-informed counseling, which augments all current counseling methodologies as recommended by best practice authorities.

The American Mental Health Counselor Association (2) sees this knowledge base as essential to practice:

“Understand the impact of various types of trauma (e.g., sexual and physical abuse, war, chronic verbal/emotional abuse, neglect, natural disasters, etc.) may have on the central nervous system (CNS) and the autonomic nervous system (ANS) and how this might impact one’s sense of secure attachment, affect regulation, personality functioning, self-beliefs and self-identity, self-care, etc., as well as the potential for trauma-related re-enactment in relationships”.

Knowledge of the neuroscience of addiction provides the counselor and the client(s) a temporal and spatial lens through which to understand addiction and its developmental course and to accelerate recovery. The process of recovery [we define as the achievement of sustained remission as specified by the Diagnostic and Statistical Manual of Mental Disorders, Fifth Edition, Text Revision (DSM-5-TR)] is associated with significant healthy neurobiological changes that invariably present emotional and decision-making challenges, which may cause relapse (3). Understanding these changes allows both the counselor and the client to set and monitor daily practices and realistic goals as neuroscience predicts. Understanding the affective and cognitive outcomes resulting from the specific elements of the brain disorder is remarkably effective in facilitating acceptance and minimizing shame for clients (4).

This article extends that union by showing how advanced artificial intelligence (AI) systems can support neurocounseling in the first months of recovery. Early recovery is a time of rapid, significant neuroplastic changes in the brain, perhaps more than most other brain disorders (5). Progress and evolving prognosis through the first year remain difficult to assess consistently and accurately using current practices, contributing to relapses among persons in care. The development of accurate metrics for continual assessment would substantially raise the bar of care methods and outcomes.

The focus is on application, learning, and integration. The goal is not to automate counseling, but to provide clinicians, peers, and clients with tools that sharpen assessment, anticipate risk, and personalize help, all with low burden and high respect for human agency. The core hypothesis is that AI can make the process of accepting Step One, and the weeks and months that follow, more predictable, measurable, and accelerate recovery without displacing the personal responsibility and community connection that benefits recovery. Development and refinement of the metrics involved in assessment may also contribute significantly to the precision of behavioral neuroscience through the involvement of large numbers of subjects at a relatively low cost of technology over time.

To provide a framework for the assessment and monitoring of clients’ progression through recovery, we borrow from the Acceptance and Commitment Therapy literature (6, 7). Acceptance is a continuous process of actively embracing the private events evoked in the past and present without unnecessary attempts to change their frequency or form, especially when doing so would cause psychological harm. From a neuroscience-informed counseling approach, acceptance also includes a process of understanding the associations among substance-induced maladaptations in the brain, cognition, emotion, and behavior.

Unmanageability refers to aspects of daily life, typically as a result of the negative consequences from addiction seeming unmanageable (8, 9). Our view is that unmanageable life circumstances arise from a broader disorder of decision making, preceding and progressive with the development of addiction (1). Acceptance and unmanageability can be seen as indices to assess and monitor progress through recovery of neurotypical brain function over time. Scales to quantify these parameters have been reported in the literature (10, 11) to guide this process.

## 2 Recent AI developments relevant to addiction recovery

Recent advances in AI have direct relevance to the assessment and care of substance use disorders.

- Large language models can interpret narratives, summarize clinical notes, and detect patterns of meaning, stance, and affect in text or speech. With careful supervision, they can help quantify constructs such as acceptance, ambivalence, or perceived unmanageability.
- Quantification scales for the assessment of acceptance and unmanageability indices through the recovery process exist in the literature and are readily adaptable to AI processes as episodic client surveys.
- Multimodal learning enables the integration of data streams such as mobile sensing, wearables, sleep and activity logs, geolocation, and brief self-reports. This supports continual, privacy-preserving risk estimation with little user effort.
- Causal machine learning that associates neurobiology and behavioral outcomes can be both exploratory and targeted to allow researchers to infer likely effects of interventions in real-world data while addressing confounding variables. These methods can complement randomized trials in complex community settings.
- Reinforcement learning with safety constraints can optimize just-in-time adaptive interventions that deliver prompts or supports when risk is high, but with safeguards that maintain therapeutic appropriateness and user autonomy.
- Federated and privacy-enhancing learning methods enable model training across institutions or communities without centralizing sensitive data, which aligns with traditions of anonymity and confidentiality in recovery settings.

These capabilities are already reshaping clinical research in mental health and behavioral medicine (12, 13). The question is how to adapt them to neurocounseling for addiction in a way that is feasible, ethical, and clinically useful.

## 3 Impact on neurocounseling practice

We propose a modular framework, i.e., AI-augmented neurocounseling for Step One (AANS-1), with four mutually reinforcing components: measurement, interpretation, intervention, and learning. Each component is designed to be optional, allowing stepwise adoption in research and practice.

### 3.1 Measurement: multimodal digital phenotyping

Early remission is dynamic. Risk fluctuates with stressors, cues, sleep, social contact, physical health, and cognitive and allostatic load. AANS-1 supports low-burden measurement through a small set of data streams, as follows:

- Brief ecological surveys of acceptance, craving, stress, commitment to abstinence, decisions made, and actions taken, delivered once or twice daily.
- Passive sensing of sleep, stress, and activity from wearables, when consented, to capture fatigue, circadian regularity, brain activity, and physical recovery.
- Geospatial exposure to high-risk locations, represented in a privacy-preserving manner using coarse geofences or on-device computation.
- Short voice journals where clients reflect on challenges and wins, with local transcription and optional encrypted upload.

The aim is not exhaustive surveillance. The goal is a compact set of signals that track key neurocognitive processes relevant to progression toward remission, such as cue reactivity, stress responsivity, and cognitive control function restoration.

### 3.2 Interpretation: indices of acceptance and unmanageability

Early recovery from addiction involves a cognitive and affective shift that many describe as honesty, surrender, or clarity about the limits of control. Acceptance, the willingness to make room for and embrace the inner unwanted experiences (14), is seen as fundamental to recovery. The literature acknowledges acceptance as a therapeutic coping process associated with higher level of life satisfaction and overall quality of life (11). Reliable scales to assess acceptance (e.g., Unconditional Self-Acceptance Questionnaire, USAQ) have been in use for decades (15) and recently updated (16). For our purposes, we adapt the USAQ to represent *A_t_*, the acceptance index, on a 0–1 scale, over time *t* (in days).

**Figure 1:**
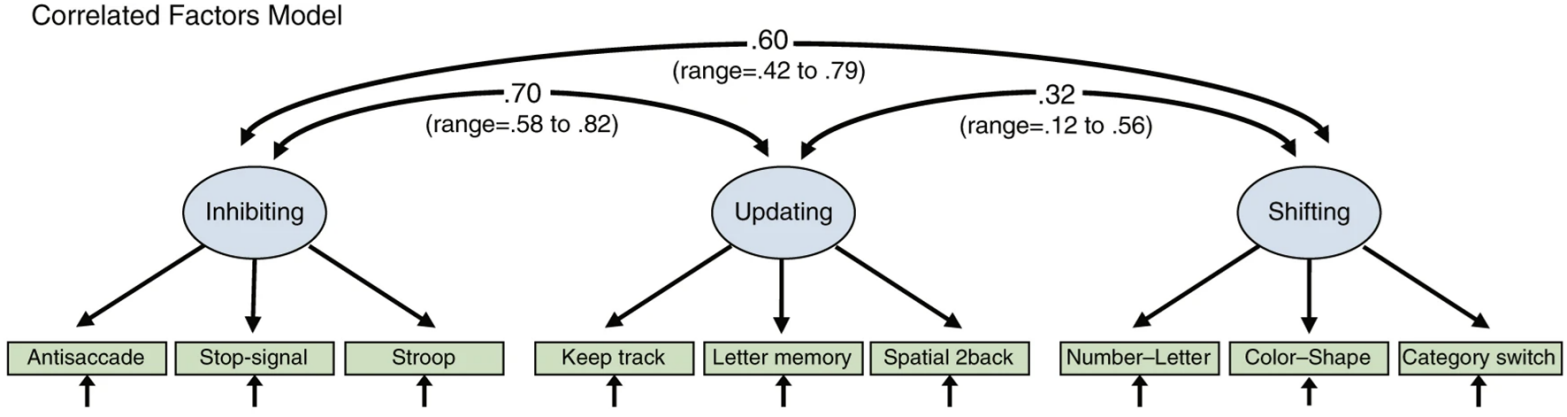
Latent factor model of cognitive control (CC). Reprinted from ‘The role of prefrontal cortex in cognitive control and executive function’ by Friedman and Robbins 2021, licensed under Creative Commons Attribution 4.0 International License, https://doi.org/10.1038/s41386-021-01132-0.

Similarly, an unmanageability index, *U_t_*, can be constructed to reflect practical instability, such as missed obligations, conflicts, or crises of varying magnitude. Unmanageability can be considered a result of cognitive control deficits (1), which can be organized according to the following control framework given by Friedman and Robbins (17):

Network-based functional restructuring of the neural mechanisms involved in cognitive control processes has been shown to underlie the cognitive impairments observed in addiction (18). Impaired decision making in addiction and early abstinence (1), leading to unmanageable life circumstances in both content and control, is multifaceted, arising from deficits in the factors shown in **Figure 1**. Recent work on the default mode network and connectivity among structures (19) supports this model for cognitive control from a neurobiological framework.

Salience attribution and attentional bias contribute from a bottom-up perspective; along with cognitive deficits, there are causal associations between addiction severity and network functional deficits (19, 20). Over the past four decades, the emergence of computer-based, valid cognitive testing programs has resulted in accessible assessment tools (21, 22). While some testing programs have corresponding associations with structural and functional findings from functional MRI (fMRI), their applications in addiction remain limited in the literature (Creyos Health).

Indeed, while there is much literature articulating the structural and functional impairments in brain regions and connectivity, “the actual pattern of diminished control observed in addiction is puzzlingly partial” (23). The frequent prevalence of relapse despite often burdensome efforts to maintain abstinence remains puzzling from any current perspective. On this basis, addiction is considered a chronic, relapsing disorder (National Institute on Drug Abuse, NIDA) for which therapies continue to evolve. While neuroscience can inform these therapies through addressing functional brain deficits, the bridge between behavior and neuroscience in the recovery brain in abstinence remains unclear (23).

The complexities and variabilities involved in the developmental nature and the dynamic scale of addiction severity and recovery progression call for advanced methods to understand the neurobiology and develop evidence-based therapies. While current therapies remain highly individualized (1), we portend that scientific knowledge on brain changes, inexpensive monitoring methods, and intelligent systems exist to support far more effective and targeted therapies. We have previously summarized the broad changes in the brain in addiction through a broad review of the literature (1). These could represent a starting point for exploring the behavioral neuroscience of recovery and assessing various treatments over time. To develop the needed initial algorithm for intelligent systems, a summary set (*E_i_*) of 10 nuclei and subnuclei in the limbic system become adversely adapted to substances coincident with stress, resulting in addiction. Adaptations as expressed in *E_i_* can be characterized as resulting from seven varied potential neuroadaptations to stress and substances. The consistent neurobiological deficits seen in addiction can be expressed as:

E_i_: Empiric evidence of deficit as reported in the literature.

Coincident with *E_i_* or sets thereof of multiple areas of deficits are consistent behavior patterns that could be initially mapped according to the latent factor model shown in **Figure 1**.

B_ij_: Behavior relationships with E_i_.

whereby *B_ij_* could represent a finite set initially defined through an accepted behavioral symptomatology in addiction (DSM 5-TR).

We portend that the correspondence between the severity of *E_i_* and *B_ij_* can be denoted by:

*R_i,j_*, where *R* is a numeric 0→1.

corresponding to the degree of deficit and the corresponding behavior, which increases over time in recovery from substances.

Unmanageability of external circumstances and emotional responses arising from addiction activates the stress response, which presents a relapse risk (24). As an initial monitoring system to wearable biomarkers for stress assessment (25–27), wearable stress sensors are often used in simple and practical applications. These sensors are friendly to the person being investigated for stress, have low complexity, and do not require cumbersome medical protocols. As wearables, these are calibrated within physiological limits and integrated over a specific time to inform an uncertainty index, *U_t_*, where *t* (time) is a daily integrated measure throughout the assessed period of recovery.

In summary, to create the initial algorithm to monitor progress in recovery over time, we used the following variables:

(*E_i_*, *B_ij_*, and *R_i,j_*) represents the correspondence among brain and behaviour as well degree of deficits.

Regular weekly cognitive testing and noninvasive monitoring methods suggested previously can create a dataset over the study time period to monitor progress and the potential effectiveness of current therapies.

In parallel, the broader milestones, i.e., acceptance and unmanageability, assessed using *A_t_* and *U_t_*, respectively, can be estimated and followed in the same time frame. Therapeutic actions are noted throughout the study period, providing an ability to better assess the effectiveness of therapies over time and the varying recovery states.

### 3.3 Learning: causal discovery and personalization

Understanding what works for whom requires careful causal reasoning. AANS-1 encourages researchers to combine:

- N-of-1 experiments, where the timing and type of micro-interventions are randomized within safe limits, yielding individual response curves.
- Targeted causal estimation to infer the average and heterogeneous effects of actions in observational data where full randomization is infeasible; and
- Digital twins, simple dynamical models of each participant’s recovery trajectory, which help simulate how different levels of therapy, meeting attendance, sleep regularity, or sponsor contact might influence risk over coming days.

The objective is not only the prediction and prevention of relapse risk but also an explanation of the mechanisms that connect neurocounseling constructs with measurable changes in daily life.

## 4 Interdisciplinary potential

AANS-1 invites collaboration across several domains.

- Clinical neuroscience can provide candidate biomarkers for stress regulation and executive function that map to study variables and help refine causal hypotheses.
- Counseling, psychology, and psychiatry can guide the definition and validation of the acceptance and unmanageability indices and ensure that AI outputs add clarity to the therapeutic process, not confusion. This field can additionally provide input into interpretability and integration into the clinical workflow.
- Behavioral economics can model commitment devices and present-bias mitigation as part of just-in-time support, e.g., prompts that align future goals with near-term choices.
- Public health can evaluate community-level deployment, including prevention of overdose, linkage to care, and equity of access.
- Peer recovery communities can co-design interfaces that respect the traditions of anonymity, mutual aid, and non-commercial support.
- Data science can advance privacy-preserving learning, interpretable models, and robust evaluation methods for real-world behavioral health. It can additionally assure data quality and mitigate potential algorithm bias as continuously embedded quality assurance processes throughout trials.

This interdisciplinary synthesis grounds AI in human practice, with each field informing not only techniques but also values and constraints.

## 5 Ethical, policy, and social implications

Ethical alignment is a first-class requirement for AI in recovery.

### 5.1 Anonymity and consent

The 12-step traditions emphasize anonymity and service. Any technological support should be optional, minimally intrusive, and separate from meeting governance. Consent must be informed, revocable, and granular, allowing users to choose which data streams to share and with whom. Federated learning and on-device inference can reduce central data collection. No system should infer participation in 12-step programs and professional counseling without explicit, repeated consent.

### 5.2 Clinical oversight and role clarity

AI suggestions must remain advisory. Counselors, physicians, and qualified peer mentors retain decision authority. Documentation should clearly attribute recommendations to an algorithmic source, including rationale and confidence. Liability frameworks should prevent either uncritical automation or defensive underuse. Training should prepare practitioners to interpret indices and negotiate recommendations with clients.

### 5.3 Fairness and access

Models trained on data from one community may not generalize. Performance must be audited across diverse populations, including differences in age, gender, ethnicity, rural *versus* urban settings, and co-occurring conditions. Access should not be dependent on expensive hardware. Community partnerships can ensure the availability of low-cost devices and offline functionality.

### 5.4 Commercial influence and community integrity

Recovery communities guard against the commercialization of service. Research groups and vendors must respect this by separating any financial incentives from program activities, avoiding targeted advertising, and publishing methods and performance transparently. Data stewardship should involve community representatives and independent oversight.

### 5.5 Safety and crisis response

Automated monitoring raises questions about duty to act. Clear protocols are needed for risk thresholds that trigger human outreach, links to crisis lines, and emergency services, with sensitivity to user preference and local law. The system should degrade gracefully when data are missing and must avoid punitive or shaming messages.

## 6 Study design and evaluation

We outline a staged research program to evaluate the AANS-1 framework.

### 6.1 Stage 1: construct validation

Recruit a small cohort beginning recovery and collect 2–4 weeks of multimodal data with high consent control. Clinicians and participants jointly label daily levels of acceptance and unmanageability. Evaluate convergent validity with counseling notes and self-report scales. Assess inter-rater reliability and continuously refine the feature sets for *E_i_*, *B_ij_*, *R_i,j_*, *A_t_*, and *U_t_*.

### 6.2 Stage 2: policy prototyping

Implement offline policy learning using de-identified trajectories. Restrict the action set to well-established supports such as call a peer, attend a counselling session or meeting, or practice a breathing exercise. Use simulated deployment to test safety constraints and present rationales that are understandable to counselors. Conduct usability sessions with practitioners and peers.

### 6.3 Stage 3: *N*-of-1 just-in-time trials

Randomize within-person timing of prompts over 8–12 weeks, stratified by current variables. Primary outcomes include day-level craving, near-term abstinence, and stress regulation. Secondary outcomes can include alliances, acceptance, perceived stress, and helpfulness. Analyze heterogeneous treatment effects to personalize policies without compromising safety.

### 6.4 Stage 4: hybrid effectiveness–implementation

Run cluster trials in collaboration with clinics and community organizations. Compare standardized care models including peer support *versus* the same with AANS-1 augmentation. Measure abstinence, quality of life, meeting participation, health service use, and relapse and overdose events over 6–12 months. Include implementation metrics such as adoption, fidelity, cost, and community acceptability.

### 6.5 Stage 5: causal discovery

Pool data with federated learning across sites to estimate how changes in acceptance and unmanageability mediate outcomes. Test hypotheses such as whether the increases in *A_t_* precede the reductions in craving after controlling for sleep regularity and peer contact. Share de-identified summaries and code for reproducibility.

## 7 From advanced AI to AGI or ASI: plausible transformations

As systems approach more general capabilities, three opportunities and three guardrails stand out.

### 7.1 Opportunities

- Mechanism discovery. More general systems could help synthesize cross-disciplinary evidence to identify mediators that link the process of recovery to neurobiological change, such as shifts in salience attribution, cognitive control, or metacognitive accuracy.
- Rich simulation. Generative agents might create realistic training environments for counselors and peers, presenting diverse client narratives and trajectories, including rare but critical scenarios such as rapid escalation of substance use after stress exposure.
- Global equity. Translation, cultural adaptation, and low-resource deployment could be automated, bringing effective support to underserved regions while respecting local practices.

### 7.2 Guardrails

- Preserve human primacy. Commitment, honesty, and relationship are human choices. AI must support, but not supplant, these.
- Protect community norms. Anonymity, non-commercial service, and mutual aid are essential features, not historical artifacts.
- Require verifiable benefit. Claims of superhuman reasoning should not bypass evaluation. Every new capability must be examined for safety, fairness, and clinical value in this domain.

## 8 Foundational implications for addiction science

Integrating AI with neuroscience-informed addiction counselling may reshape core questions.

- Measurement of insight. If acceptance can be measured in ways that predict outcomes, we can move beyond debates about spiritual *versus* medical models toward a shared construct that guides care.
- Dynamic models of remission. Daily state estimates and digital twins could refine theories of early remission, capturing feedback between sleep, cognition, social contact, neurobiology, and craving.
- Personalization with humility. The combination of N-of-1 designs and community governance can challenge oversimplified universal models while avoiding fragmented hyper-personalization that ignores common therapeutic factors. These advances can make the field more cumulative, connecting phenomenology from narratives and events with measurable processes in brain and behavior.

## 9 Limitations and open questions

AANS-1 is ambitious and faces constraints.

- Data burden and engagement are real. Even minimal sensing can feel intrusive during early recovery. Co-design with users is essential.
- Language models risk misinterpretation of personal narratives. High-quality clinical supervision, bounded outputs, and clear fallbacks are needed.
- Causal conclusions will remain uncertain without careful design. Mixed methods that integrate qualitative insights with quantitative inference are required.
- Community heterogeneity is wide. What fits one person or community recovery group may not fit others. Adaptation and local governance must be built in. These challenges are tractable if addressed early and with humility.

## 10 Future outlook

In the near term, the priority is a small set of reliable tools that raise the floor of care. These include robust risk tracking, interpretable indices that help focus sessions, choice of counseling modalities, and safe just-in-time suggestions that connect clients to human supports. In the medium term, integrated datasets and federated models can clarify mechanisms and improve personalization. In the long term, if more general intelligent systems emerge, they may help unify neurobiological and psychosocial accounts of recovery. Even then, the center of gravity should remain with human judgment and community practice. AI can accelerate discovery and widen access, but it cannot replace meaning, commitment, and belonging.

## 11 Conclusion

The behavioral neuroscience underlying addiction and early recovery suggests that early commitment to change is both measurable and malleable. Intelligent systems can serve neurocounseling by sensing risk with low burden, interpreting acceptance and unmanageability with transparency, and offering timely, human-centered support. The AANS-1 framework provides a research and implementation roadmap that is faithful to the realities of clinical care and community recovery. Its success is dependent on interdisciplinary collaboration, rigorous evaluation, and ethical guardrails that respect the values of the people and communities it aims to serve. Aligning AI progress with these domain-specific needs can advance both scientific understanding and lived recovery.

## Data Availability

The original contributions presented in the study are included in the article/supplementary material. Further inquiries can be directed to the corresponding author.
